# RIC *versus* MAC UCBT in adults with AML: A report from Eurocord, the ALWP and the CTIWP of the EBMT

**DOI:** 10.18632/oncotarget.9599

**Published:** 2016-05-26

**Authors:** Frédéric Baron, Annalisa Ruggeri, Eric Beohou, Myriam Labopin, Guillermo Sanz, Noel Milpied, Mauricette Michallet, Andrea Bacigalupo, Didier Blaise, Jorge Sierra, Gérard Socié, Jan J. Cornelissen, Christoph Schmid, Sebastian Giebel, Norbert-Claude Gorin, Jordi Esteve, Fabio Ciceri, Bipin N. Savani, Mohamad Mohty, Eliane Gluckman, Arnon Nagler

**Affiliations:** ^1^ University of Liege, Liege, Belgium; ^2^ Eurocord, Hospital Saint Louis, AP-HP, and IUH University Paris VII, Paris, France; ^3^ AP-HP, Hématologie Clinique et Thérapie Cellulaire, Hôpital Saint-Antoine, Paris, France; ^4^ EBMT Paris Office, Hospital Saint Antoine, Paris, France; ^5^ Hospital Universitario La Fe, Servicio de Hematologia, Valencia, Spain; ^6^ CHU Bordeaux, Hématologie Clinique et Thérapie Cellulaire-Hôpital Haut-leveque, Bordeaux, France; ^7^ University of Bordeaux, Bordeaux, France; ^8^ Service d'Hematologie du Centre Hospitalier de Lyon Sud, Pierre-Bénite, France; ^9^ Ospedale San Martino, Department of Haematology II, Genova, Italy; ^10^ Institut Paoli Calmettes (IPC), Aix Marseille University (AMU), UM105, Centre de Recherche en Cancerologie (CRCM), Inserm U1068, CNRS UMR7258 Marseille, Marseille, France; ^11^ Hospital Santa Creu i Sant Pau, Hematology Department, Barcelona, Spain; ^12^ AP-HP, Hematology Transplantation, Hospital Saint-Louis, Paris, France; ^13^ Erasmus Medical Center-Daniel den Hoed Cancer Center, Rotterdam, The Netherlands; ^14^ Klinikum Augsburg, Department of Hematology and Oncology, University of Munich, Augsburg, Germany; ^15^ Maria Sklodowska-Curie Cancer Center and Institute of Oncology, Gliwice Branch, Gliwice, Poland; ^16^ Deptartment of Hematology, Hospital Clinic, Barcelona, Spain; ^17^ Department of Hematology, Ospedale San Raffaele, Università degli Studi, Milano, Italy; ^18^ Long Term Transplant Clinic, Vanderbilt University Medical Center, Nashville, TN, USA; ^19^ Universite Pierre & Marie Curie, Paris, France; ^20^ INSERM, UMRS 938, Paris, France; ^21^ Eurocord, Hospital Saint Louis, AP-HP, and IUH University Paris VII, France Monacord, Centre Scientifique de Monaco, Monaco; ^22^ Division of Hematology and Bone Marrow Transplantation, The Chaim Sheba Medical Center, Tel-Hashomer, Ramat-Gan, Israel

**Keywords:** unrelated cord blood, AML, reduced-intensity, myeloablative, transplantation

## Abstract

Nonrelapse mortality (NRM) is the first cause of treatment failure after unrelated cord blood transplantation (UCBT) following myeloablative conditioning (MAC). In the last decade, reduced-intensity conditioning (RIC) regimens have been developed with the aim of reducing NRM and allowing older patients and those with medical comorbidities to benefit from UCBT. The aim of the current retrospective study was to compare transplantation outcomes of acute myeloid leukemia (AML) patients given UCBT after either RIC or MAC. Data from 894 adults with AML receiving a single or double UCBT as first allograft from 2004 to 2013 at EBMT centers were included in this study. 415 patients were given UCBT after RIC while 479 patients following a MAC. In comparison to MAC recipients, RIC recipients had a similar incidence of neutrophil engraftment and of acute and chronic graft-versus-host disease (GVHD). However, RIC recipients had a higher incidence of disease relapse and a lower NRM, translating to comparable leukemia-free (LFS), GVHD-free, relapse-free survival (GRFS) and overall survival (OS). These observations remained qualitatively similar after adjusting for differences between groups in multivariate analyses. In conclusion, these data suggest that LFS and OS are similar with RIC or with MAC in adults AML patients transplanted with UCBT. These observations could serve as basis for a future prospective randomized study.

## INTRODUCTION

Allogeneic hematopoietic stem cell transplantation (allo-HCT) from HLA-identical sibling is the treatment of choice for selected patients with acute myeloid leukemia (AML) [1–3]. For AML patients who lack a suitable human leukocyte antigen (HLA)-identical sibling, unrelated cord blood transplantation (UCBT) is an adequate alternative to HLA-matched unrelated bone marrow/peripheral blood stem cell (PBSC) transplantation, particularly for patients at high risk of rapid disease relapse who urgently need a transplantation [4, 5].

Despite major improvements in the field, nonrelapse mortality (NRM) has remained the main cause of failure of UCBT for AML [5]. In the last decade, reduced-intensity conditionings (RIC) for UCBT have been developed with the aim of reducing NRM and allowing older/unfit patients to benefit from UCBT [6–9]. Although a recent study demonstrated low NRM after RIC UCBT for AML [10], a high incidence of disease relapse has also been observed with this approach [10–12]. This prompted us to perform the current retrospective registry study aimed at assessing the impact of the conditioning intensity on transplantation outcomes in patients receiving UCBT as treatment for AML.

## RESULTS

### Patient, disease and transplant characteristics

Patients and disease characteristics are described in Table 1. Briefly, 415 patients were given UCB after RIC while 479 patients were administered MAC. The most frequently used conditioning regimens were either TCF regimen, given in 18% of MAC recipients and 74% of RIC recipients, respectively, or TBF given in 39% of MAC recipients and 5% of RIC recipients. In comparison to MAC recipients, RIC recipients were almost 2 decades older [median age 54 (range, 19 - 72) years *versus* 37 (range, 18 - 68) years, *P* < 0.001], received more frequently a double UCBT (62% *versus* 29%, *P* < 0.001), received more frequently units with > or = 2 HLA-mismatches (67% *vs* 56%, *P* = 0.003), received higher TNC [median 3.5 (range, 0.3 - 11.8) 10E7 cells/kg *versus* 2.8 (range, 0.2 - 40.3) 10E7 cells/kg, *P* < 0.0001], and received less frequently ATG (23% *versus* 60%, *P* < 0.0001) in the conditioning. Disease status at UCBT was comparable in both groups with approximately half of the patients in first CR and 19% of patients not in CR in both groups. Median follow-up for survivors was 26 (range, 1.02 −118.2) months.

**Table 1:** Patient and transplant characteristics

	MAC (*n* = 479)	RIC (*n* = 415)	*P* value ^1^
**Median patient age, y (range)**	37 (18 – 68)	54 (19-72)	<0.0001
**Median year of UCBT, y (range)**	2010 (2004-2013)	2010 (2004-2013)	0.16
**Recipient gender M, # (%)**	251 (52)	185 (45)	0.02
**Status at transplantation, # (%)**			
CR1	248 (52)	200 (48)	0.06
CR2	123 (26)	131 (32)	
CR3	19 (4)	7 (2)	
Advanced	89 (19)	77 (19)	
**Cytogenetics, # (%) all patients**			0.0004
Good risk^2^	42 (9)	20 (5)	
Intermediate risk^3^	221 (46)	229 (55)	
High risk^4^	50 (10)	61 (15)	
Not reported/failed	166 (35)	105 (25)	
**Cytogenetics, # (%) patients in CR1**			
Good risk^2^	10 (4)	0 (0)	
Intermediate risk^3^	122 (49)	111 (56)	
High risk^4^	29 (12)	41 (20)	
Not reported/failed	87 (35)	48 (24)	
Normal cytogenetics and FLT3-ITD+	25 (11)	28 (12)	
Missing	71 (30)	60 (26)	
**Conditioning regimen, # (%)**			
TCF^5^	85 (18)	308 (74)	<0.0001
TBF^6^	176 (37)	21 (5)	
BuCy	41 (9)	5 (1)	
BuFlu	40 (8)	8 (2)	
FluMel	4 (1)	15 (4)	
TreoFlu	7 (1)	4 (1)	
TBI-based but not TCF	82 (17)	29 (7)	
Others	44 (9)	25 (6)	
**Recipient CMV-seronegative, # (%)**	287 (71)	255 (64)	0.06
**Cord blood, # (%)**			
Single	341 (71)	159 (38)	<0.0001
Double	138 (29)	256 (62)	
**ATG, # (%)**	267 (60)	89 (23)	<0.0001
**TNC at infusion x 10^7^/kg**			
Median (range)	2.8 (0.2-40.3)	3.5 (0.3-11.8)	<0.0001
Missing data (# of patients)	127	82	
**Number of HLA disparities, # (%)**			
0-1 mismatch	166 (44)	109 (33)	0.003
2-4 mismatches	208 (56)	217 (67)	
Missing data	105	89	
**Postgrafting immunosuppression, # (%)**			<0.0001
CSP ( or tacro) alone	46 (10)	22 (6)	
CSP (or tacro) + MMF	218 (49)	335 (86)	
CSP + MTX	24 (5)	11 (3)	
CSP + MMF + MTX	6 (1)	3 (1)	
Post-transplant cy	8 (2)	4 (1)	
Other	142 (32)	14 (4)	
Missing	35	26	

MAC, myeloablative conditioning; RIC, reduced-intensity conditioning; Y, year; M, male; UCBT, umbilical cord blood transplantation; CR, complete remission; #, number of patients; ATG, anti-thymocyte globulins; TNC, total nucleated cells; tacro, tacrolimus; CSP, cyclosporine A; MMF, mycophenolate mofetil. TCF, total body irradiation (TBI), cyclophosphamide and fludarabine; TBF, Thiothepa, busulfan and fludarabine.

1 calculated with c^2^ statistics for categorical variables and Mann-Whitney test for continuous variables;

2 defined as t(8;21), t(15;17), inv or del (16), or acute promyelocyticleukemia, these abnormalities only or combined with others;

3 defined as all cytogenetics not belonging to the good or high risk (including trisomias);

4 defined as 11q23 abnormalities, complex karyotype, abnormalities of chromosomes 5 and 7;

5 classified as RIC if the dose of TBI was < 6 Gy;

6 classified as RIC when the busulfan total dose was ≤ 8 mg/kg).

### Engraftment and GVHD

Overall, CI of neutrophil engraftment at day 100 was not different in RIC (89%) and MAC (88%) recipients (*P* = 0.8) with the limitation that we did not systematically collect chimerism data and that autologous reconstitution is possible in RIC recipients. Median times for reaching 0.5 × 10^9^/L neutrophils were 21 (range, 3-66) days in RIC patients *versus* 23 (range, 1-106) days in MAC patients, respectively.

In univariate analysis, there was a higher incidence of grade II-IV acute GVHD in RIC recipients (35% *versus* 26%, *P* = 0.009) while the incidence of grade III-IV acute GVHD was similar in both groups of patients (11% and 11%, *P* = 0.9). However, in multivariate analyses adjusting for single *versus* double UCBT, gender combination, use of ATG and HLA-compatibility the incidence of grade II-IV acute GVHD was comparable in RIC and MAC recipients (HR = 1.09, *P* = 0.65).

The 2-year cumulative incidence of chronic GVHD was similar in RIC and MAC recipients (23% and 23%, *P* = 0.9). In multivariable analysis, there was a trend for a higher incidence of chronic GVHD in patients receiving the TBF regimen [HR 1.7 (95% CI, 1.0-2.7), *P* = 0.05], while, interestingly, ATG failed to decrease the incidence of chronic GVHD [HR 1.4 (95% CI, 0.9-2.2), *P* = 0.15].

### Relapse, NRM, LFS, OS

**Figure 1:**
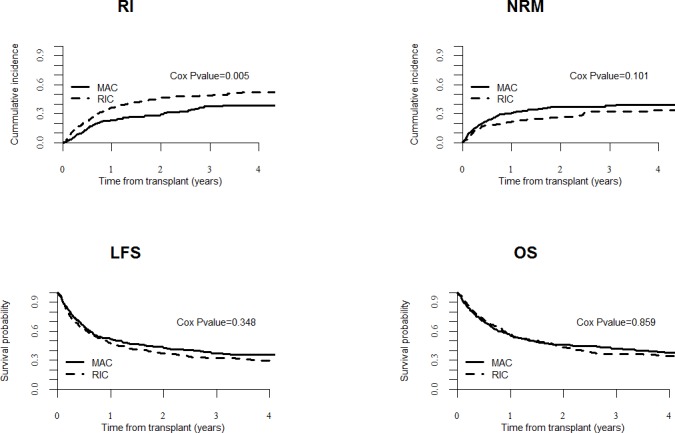
UCBT outcomes in AML patients transplanted following RIC (*n* = 415) *versus* MAC (*n* = 479) The figures show the unadjusted curves for MAC patients and the adjusted curves for RIC recipients. Curves were adjusted for age at transplantation, recipient gender, year of transplantation, disease status, TBF conditioning or not, TCF conditioning, or not, and the use of ATG. LFS, leukemia-free survival; OS, overall survival; RI, relapse incidence and NRM, nonrelapse mortality.

At 2-year, RIC recipients had a higher incidence of disease relapse (41% *versus* 23%, *P* < 0.001) but a lower NRM (19% *versus* 36%, *P* < 0.001), translating to similar LFS (40% *versus* 41%, *P* = 0.8) and OS (46% *versus* 43%, *P* = 0.3) than when compared to MAC recipients (Figure 1). In multivariate analyses, the use of RIC (*versus* MAC) regimen was associated with a higher incidence of relapse (HR = 1.6, 95% CI: 1.2-2.2; *P* = 0.005). LFS and OS were comparable (LFS: HR = 1.1, 95% CI:0.9-1.4; *P* = 0.3); (OS: HR = 1.0, 95% CI:0.8-1.3; *P* = 0.9) (Table 2). Factors associated with worse OS included older patient age (HR = 1.0, 95% CI: 1.0-1.0; *P* = 0.02), advanced disease (HR = 2.3, 95% CI: 1.8-2.9; *P* < 0.0001), while female recipients had better OS than male recipients (HR = 0.8, 95% CI:0.7-1.0; *P* = 0.02).

**Table 2:** Multivariate analyses

		*P* value	HR	Lower	Upper
Relapse or death	RIC *vs* MAC	.348	1.1	0.9	1.4
	Age at tx (in years)	.090	1.0	1.0	1.0
	**Female** ***vs*** **Male**	**.019**	**0.8**	**0.7**	**1.0**
	Year of tx	.816	1.0	1.0	1.0
	CR2 *vs* CR1	.257	1.1	0.9	1.4
	**Advanced** ***vs*** **CR1**	**.000**	**2.2**	**1.7**	**2.7**
	Double *vs* Single	.436	0.9	0.7	1.1
	TCF used	.166	0.8	0.6	1.1
	TBF used	.426	0.9	0.7	1.2
	ATG used	.061	1.3	1.0	1.6
Death	RIC *vs* MAC	.859	1.0	0.8	1.3
	**Age at tx (in years)**	**.017**	**1.0**	**1.0**	**1.0**
	**Female** ***vs*** **Male**	**.023**	**0.8**	**0.7**	**1.0**
	Year of tx	.731	1.0	1.0	1.0
	CR2 *vs* CR1	.117	1.2	1.0	1.5
	**Advanced** ***vs*** **sCR1**	**.000**	**2.3**	**1.8**	**2.9**
	Double *vs* Single	.273	0.9	0.7	1.1
	TCF used	.221	0.8	0.6	1.1
	TBF used	.812	1.0	0.7	1.3
	ATG used	.085	1.2	1.0	1.6
RI	**RIC** *vs* **MAC**	**.005**	**1.6**	**1.2**	**2.2**
	Age at tx (in years)	.794	1.0	1.0	1.0
	Female *vs* Male	.354	0.9	0.7	1.1
	Year of tx	.552	1.0	0.9	1.0
	CR2 *vs* CR1	.632	0.9	0.7	1.3
	**Advanced** ***vs*** **CR1**	**.000**	**3.2**	**2.4**	**4.4**
	Double *vs* Single	.646	1.1	0.8	1.4
	TCF used	.417	0.9	0.6	1.2
	TBF used	.100	0.7	0.4	1.1
	ATG used	.841	1.0	0.7	1.3
NRM	RIC *vs* MAC	.101	0.7	0.5	1.1
	**Age at tx (in years)**	**.018**	**1.0**	**1.0**	**1.0**
	**Female** ***vs*** **Male**	**.026**	**0.7**	**0.6**	**1.0**
	Year of tx	.925	1.0	0.9	1.1
	**CR2** ***vs*** **CR1**	**.037**	**1.4**	**1.0**	**1.8**
	Advanced *vs* CR1	.090	1.4	1.0	2.0
	Double *vs* Single	.110	0.8	0.5	1.1
	TCF used	.236	0.8	0.5	1.2
	TBF used	.867	1.0	0.7	1.5
	**ATG used**	**.012**	**1.6**	**1.1**	**2.3**

HR, hazard ratio; CI, confidence interval; P, *p* value; cGVHD, cumulative incidence of chronic graft-versus-host disease; NRM, cumulative incidence of non relapse mortality; RI, cumulative incidence of relapse; LFS, leukemia-free survival; OS, overall survival; Tx, transplantation; CR, complete remission; advanced, not in complete remission; ATG, anti-thymocyte globulin; RIC, reduced-intensity conditioning; MAC, myeloablative conditioning.

### GRFS

GVHD-free, relapse-free survival (GRFS) has recently emerged as an important endpoint in allo-HCT. We thus compared GRFS in patients receiving UCBT after RIC or MAC. At 2-year, GRFS was similar in RIC and MAC recipients (30.9% *versus* 31.1%, *P* = 0.86). In multivariate analysis, GRFS was similar in RIC and MAC patients (HR = 1.0, 95% CI: 0.8-1.3; *P* = 0.7). Factors associated with worse GRFS included advanced disease (HR 1.8, 95% CI: 1.5-2.2, *P* < 0.001) while female recipients had better GRFS than male recipients (HR = 0.8, 95% CI:0.6-0.9; *P* = 0.001).

### Additional Cox analyses for OS and LFS

**Figure 2:**
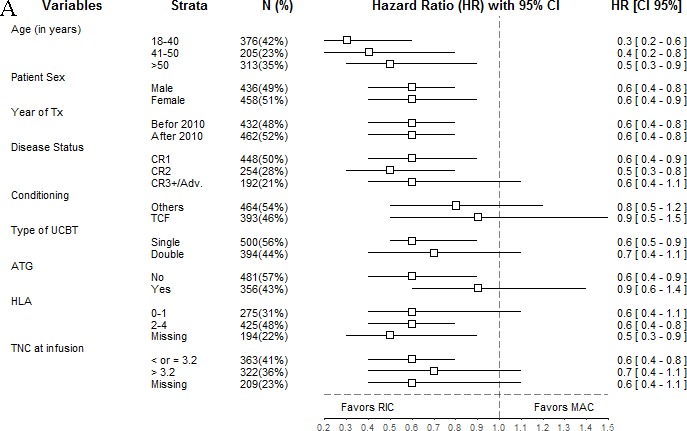

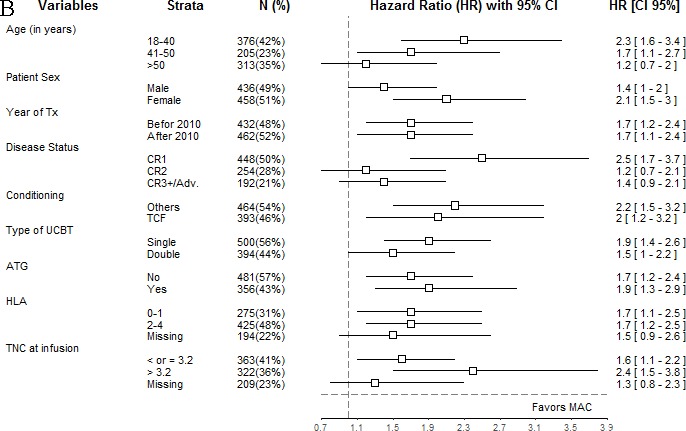
Forest plot analysis of cumulative incidence of nonrelapse mortality (**A**) and relapse (**B**) HR and 95% confidence intervals were computed using univariate Cox analyses

**Figure 3:**
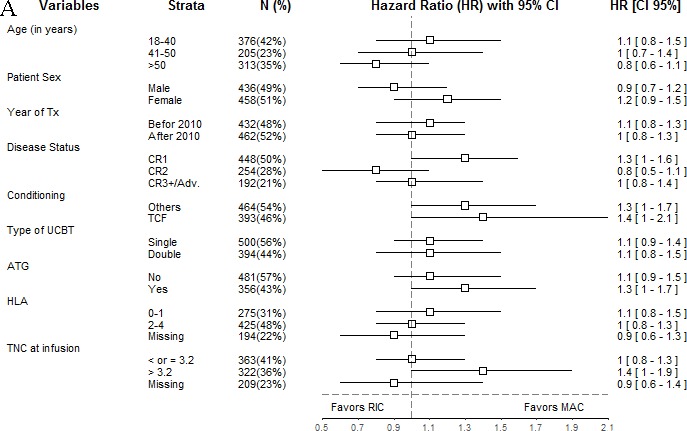

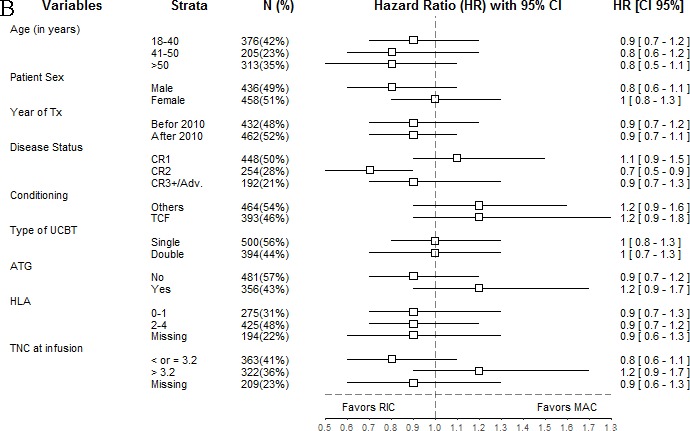
Forest plot analysis of leukemia-free survival (A) and overall survival (B) HR and 95% confidence intervals were computed using univariate Cox analyses.

To further dissect the impact of conditioning intensity on UCBT, we performed additional Cox analyses comparing UCBT outcomes among patients conditioned with RIC or MAC regimen separately for pre-transplant variables. The results of these analyses are presented graphically using Forest plots in Figures 2–3.

RIC regimens were associated with lower NRM in each subgroup categories with median HR ranging from 0.3 to 0.9 (Figure 2A). This was particularly the case in the subgroup of younger patients (HR = 0.3, 95% CI: 0.2-0.6) and in those not given ATG (HR = 0.6, 95% CI: 0.4-0.9). However, RIC regimen was also associated with a higher risk of relapse in each subgroup categories with HR ranging from 1.2 to 2.5 (Figure 2B). Interestingly, the association between RIC regimen and higher risk of relapse was not less pronounced in patients in CR1 at transplantation (HR = 2.5, 95% CI: 1.7-3.7) than in those in CR3+ or advanced disease (HR = 1.4, 95% CI HR 0.9-2.1), and was also observed in the subgroup of patients transplanted following TCF regimen (HR = 2.0, 95% CI 1.2-3.2).

As shown in Figure 3A, RIC and MAC patients had comparable LFS in most transplantation variable subgroup. Interestingly, the assessment of heterogeneity according to age group evidenced a I^2^ = 4.3%, demonstrating a very low impact of age on the association between conditioning intensity and LFS. However, there was a suggestion for worse LFS with RIC in the subgroup of patients transplanted in first CR (HR = 1.3, 95% CI: 1.0-1.6), in the subgroup of patients given ATG (HR = 1.3, 95% CI: 1.0-1.7), and in patients infused with > 3.2 x10^7^ TNC/Kg (HR = 1.4, 95% CI: 1.0-1.9). Further, interestingly, when patients were stratified according to the type of conditioning regimen used, there was a suggestion for worse LFS with RIC both in patients conditioned with the TCF regimen (HR = 1.4, 95% CI: 1.0-2.1), and in patients conditioned with other regimens (HR = 1.3, 95% CI: 1.0-1.7).

Finally, RIC and MAC patients had comparable OS in each transplantation variable subgroup (Figure 3B).

## DISCUSSION

The impact of dose intensity on outcomes in AML patients has been the focus of many studies since the development of non-myeloablative/RIC regimens in the last 2 decades [13–18]. These studies have focussed mainly on patients given PBSC as stem cell source. Large registry studies observed that the use of RIC regimen was associated with a higher risk of relapse, but also a lower incidence of NRM translating to similar OS and LFS [13–16]. More recently, the BMT-CTN performed a randomized study comparing RIC *versus* MAC in patients with MDS (*N* = 54) or AML (*N* = 218) (18-65 years, HCT-specific comorbidity index score [19] ≤ 4) who had < 5% marrow myeloblasts at allo-HCT [20]. As observed in registry studies, the use of RIC regimen was associated with higher risk of relapse (48 *versus* 14% at 18 months, *P* < 0.01) but also lower NRM (4% *versus* 16% at 18-months, *P* = 0.02). However, because the incidence of NRM was already low in MAC recipients, the amplitude of the reduction of NRM with RIC was insufficient to offset its negative impact on relapse, translating to significantly worse LFS in RIC recipients (47% *vs* 68% at 18 months, *P* < 0.01).

In contrast to allo-HCT using other stem cell source where relapse is usually the first cause of transplant failure in AML patients, NRM has remained the leading cause of death in UCB recipients given myeloablative conditioning [21]. Further, recent findings in humanized mice suggest that graft-*versus*-tumor effects might be higher with UCB than with PBSC [22], confirming prior clinical observations [23]. This could suggest that dose intensity might be less important following UCBT than following PBSCT in regards to risk of relapse. Based on these findings, we hypothesized that the use of RIC *versus* MAC might be beneficial in the UCBT setting.

This prompted us to perform a retrospective study in the EBMT/Eurocord registries comparing UCBT outcomes in patients administered a RIC or a MAC regimen. Several observations were made.

First, despite prior evidence of strong graft-*versus*-tumor effects after UCBT [23], RIC UCBT recipients had a significantly higher risk of relapse than MAC UCBT recipients (HR = 1.6, 95% CI: 1.2-2.2). This confirms the results of a prior single center study [24] and demonstrates the importance of dose intensity for preventing AML relapse even in the setting of UCBT. As expected, NRM was lower after RIC than after MAC UCBT. As a net result, LFS, GRFS and OS were comparable in RIC and MAC recipients, rejecting our hypothesis that RIC regimens would provide better outcomes than MAC in AML patients undergoing UCBT.

The current study also confirmed a detrimental impact of ATG on NRM as recently reported in a study including data from patents given UCB after MAC conditioning [25] or RIC in the double UCBT setting [26]. Further, despite ATG not only induces *in vivo* T-cell depletion of the graft but also promotes the generation of regulatory T cells [27, 28], ATG failed to prevent GVHD in the current study. However ATG had no impact either on relapse incidence, in agreement with recent observations in the PBSC transplantation setting [29, 30]. Further, as previously observed in the UCB setting [5, 11], older age was associated with worse LFS and OS.

There are some limitation in our study including its design (retrospective registry survey), and the imbalance of the two groups for risk factors known to be associated with outcome: RIC patients were almost 2 decades older and were more often given UCB with ≥2 HLA-mismatches, but they received more often double UCBT and consequently received more cells while they were given less frequently ATG. These differences were carefully adjusted for in multivariate analysis while where forest plots demonstrated comparable OS with RIC and with MAC in each pre-transplant subgroups. Interestingly, a trend for better LFS was observed in MAC recipients in the subgroup of patients transplanted in first CR, in those who received ATG, and in patients infused with > 3.2 x10^7^ TNC/Kg.

In summary, we observed that LFS and OS were similar with RIC and with MAC in adult AML patients offered UCBT. These observations could serve as basis for a future prospective randomized study. In the meantime, recent advances in the field of UCBT such as optimization of myeloablative regimen for UCBT, expansion of UCB hematopoietic stem/progenitor cells, and post-transplant administration of chimeric antigen receptor T cells are likely to improve outcomes of UCBT both in the RIC and in the MAC setting [31–33].

## PATIENTS AND METHODS

### Data collection

This survey is a retrospective study performed by the Acute Leukemia Working Party (ALWP) of the European group for Blood and Marrow Transplantation (EBMT) and by Eurocord. EBMT registry is a voluntary working group of more than 500 transplant centers, participants of which are required once a year to report all consecutive stem cell transplantations and follow-up. Eurocord collects data on UCBT performed in > 50 countries worldwide and > 500 transplant centers, mainly EBMT centers. Population selection criteria included adult recipients (defined as ≥ 18 years old at UCBT), primary AML, first allogeneic stem cell transplantation, single or double unit UCBT performed from 2004 to 2013. Grading of acute and chronic GVHD was performed using established criteria [34]. HLA-compatibility was based on antigenic level typing for HLA-A and -B, and allele-level typing for HLA-DRB1. For the purpose of this study, all necessary data were collected according to EBMT and Eurocord guidelines. RIC was defined as use of fludarabine (Flu) associated with < 6 Gy total-body irradiation (TBI), or busulfan ≤ 8 mg/kg, melphalan ≤ 140 mg/m^2^ or other nonmyeloablative drugs, as previously reported [14, 35–37]. Specifically, the combination of total body irradiation, cyclophosphamide and fludarabine (TCF regimen) was classified as RIC when the TBI dose was < 6 Gy (most RIC patients were given 2 Gy TBI) and as MAC when the TBI dose was ≥ 6 Gy (most MAC patients were given > 10 Gy TBI). Similarly, the association of thiothepa, busulfan and fludarabine (TBF regimen) was classified as RIC or MAC based on the dose of busulfan received (≤ 8 mg/kg or > 8 mg/kg, respectively).

### Ethics

The scientific boards of the ALWP of EBMT and of Eurocord approved this study.

### Statistical analyses

Data from all patients meeting the inclusion/exclusion criteria were included in the analyses. Start time was date of transplant for all endpoints. Neutrophil engraftment was defined as first of 3 consecutive days with a neutrophil count of at least 0.5 × 10^9^/L.

To evaluate the relapse incidence, patients dying either from direct toxicity of the procedure or from any other cause not related to leukemia were censored. NRM was defined as death without experiencing disease recurrence. Patients were censored at the time of relapse or of the last follow-up. Cumulative incidence functions (CIF) were used for relapse incidence and NRM in a competing risk setting, since death and relapse were competing together.

For estimating the cumulative incidence of chronic GVHD, death was considered as a competing event. OS and LFS were estimated using the Kaplan-Meier estimates. GVHD-free, relapse-free survival (GRFS) was defined as being alive with neither grade III-IV acute GVHD, severe chronic GVHD nor disease relapse [38]. Univariate analyses were done using Gray's test for CIF and log rank test for OS and LFS. Associations of patient and graft characteristics with grade II-IV acute GVHD were evaluated using multivariate logistic regression. Variables introduced in the multivariate logistic regression included conditioning intensity (RIC *versus* MAC), single or double UCBT, gender combination, use of ATG and HLA-compatibility. Associations of patient and graft characteristics with other outcomes (chronic GVHD, relapse, NRM, LFS and OS) were evaluated in multivariable analyses, using Cox proportional hazards. Variables introduced in the Cox models included conditioning intensity (RIC *versus* MAC), conditioning type (thiotepa, busulfan and fludarabine (TBF) *versus* other and TBI, Flu and cyclophosphamide (TCF) *versus* other), the use of ATG or not, recipient age, recipient gender, years of transplantation and disease status at transplantation. Exploratory analyses of the heterogeneity of RIC *vs* MAC among pre-transplant subgroups for OS and LFS were performed using Cox models. The results of these Cox models were presented graphically using forest plots [39]. Heterogeneity according to age group for LFS was assessed by calculating the I^2^ = (Qstatistic-degre of freedom)/Qstatistic x 100.

All tests were two sided. The type I error rate was fixed at 0.05 for determination of factors associated with time to event outcomes. Statistical analyses were performed with SPSS 19 (SPSS Inc, Chicago, IL), and R 2.13.2 (R Development Core Team, Vienna, Austria) software packages.
